# Harnessing Macrophages in Cancer Therapy: from Immune Modulators to Therapeutic Targets

**DOI:** 10.7150/ijbs.106275

**Published:** 2025-02-26

**Authors:** Huabing Tan, Meihe Cai, Jincheng Wang, Tao Yu, Houjun Xia, Huanbin Zhao, Xiaoyu Zhang

**Affiliations:** 1 Department of Infectious Diseases, Hepatology Institute, Renmin Hospital, Shiyan Key Laboratory of Virology, Hubei University of Medicine, Shiyan, Hubei Province, China.; 2 General internal medicine, Wuhan Jinyintan Hospital, Tongji Medical College of Huazhong University of Science and Technology, Wuhan, China.; 3 Department of Traditional Chinese Medicine, Zhushan Renmin Hospital, Zhushan, 442200, China.; 4 Faculty of Medicine, Hokkaido University, Japan.; 5 CAS Key Laboratory of Tissue Microenvironment and Tumor, Shanghai Institute of Nutrition and Health, University of Chinese Academy of Sciences, Chinese Academy of Sciences, Shanghai, China.; 6 Center for Cancer Immunology, Institute of Biomedicine and Biotechnology, Shenzhen Institute of Advanced Technology, Chinese Academy of Sciences, Shenzhen, China.; 7 Department of Pathophysiology, Key Laboratory of Cell Differentiation and Apoptosis of Chinese Ministry of Education, Shanghai Jiao Tong University School of Medicine, Shanghai, China.; 8 Present: Division of Pharmaceutical Sciences, Department of Pharmacy and Pharmaceutical Sciences, St. Jude Children's Research Hospital, Memphis, TN, USA.; 9 Department of Gastrointestinal Surgery, Huai'an Second People's Hospital, The Affiliated Huai'an Hospital of Xuzhou Medical University, Huai'an, China.

**Keywords:** macrophage, cancer immunity, immunotherapy, phagocytotic checkpoint, trained macrophage

## Abstract

Macrophages, as the predominant phagocytes, play an essential role in pathogens defense and tissue homeostasis maintenance. In the context of cancer, tumor-associated macrophages (TAMs) have evolved into cunning actors involved in angiogenesis, cancer cell proliferation and metastasis, as well as the construction of immunosuppressive microenvironment. Once properly activated, macrophages can kill tumor cells directly through phagocytosis or attack tumor cells indirectly by stimulating innate and adaptive immunity. Thus, the prospect of targeting TAMs has sparked significant interest and emerged as a promising strategy in immunotherapy. In this review, we summarize the diverse roles and underlying mechanisms of TAMs in cancer development and immunity and highlight the TAM-based therapeutic strategies such as inhibiting macrophage recruitment, inhibiting the differentiation reprogramming of TAMs, blocking phagocytotic checkpoints, inducing trained macrophages, as well as the potential of engineered CAR-armed macrophages in cancer therapy.

## 1. Introduction

Tumorigenesis is a process of normal cells being transformed into cancer cells and characterized by uncontrolled tumor cell growth and impaired immune surveillance. The development and progression of tumors are influenced by a variety of factors. Primarily, oncogenic mutations and the activation of signaling pathways driven by these mutations play a key role 1-5. Additionally, the interaction between tumor cells and the surrounding microenvironment significantly contributes to tumor growth. The tumor microenvironment (TME), a dynamic and complex milieu of various stromal cells around cancer cells, plays a critical role in tumor progression and treatment efficacy 6-10. Tumor-associated macrophages (TAMs) are observed as the most abundant infiltrated immune cells in the TME 11. As is known, macrophages are critical for inflammation, tissue repair, organ regeneration, and tissue homeostasis. By secreting growth factors, proteases, and cytokines, TAMs interact with other cell populations within tumors and are involved in pro-tumorigenic or anti-tumorigenic roles in various cancers 12, 13. TAMs are extremely heterogeneous in TME which are determined by their ontogeny, intrinsic factors, and locations 14. Throughout the different stages of malignant cancer, the sub-populations of TAMs are dynamically changed and are programed to increasingly adopt immune suppressive characteristics along with the tumor progression. The expansion of TAMs accelerates the formation of immunosuppressive TME driven by self-proliferation and monocyte differentiation 15. In addition, tissue resident macrophages (TRMs) foster an anti-inflammatory conditions in organs which provide ideal niches for promoting metastasis, for example, peritoneal GATA6^+^ TRMs promote the ovarian cancer metastasis into the peritoneal cavity 16, 17 and liver 9. Moreover, TAMs impede the CD8^+^ T cell mediated anti-tumor immune response, which is typically boosted by immune checkpoint blocking (ICB) 18, 19. In summary, these data underscore the significant involvement of TAMs highly in shaping of the context of cancers during tumorigenesis.

With the application and innovation of multi-omics, more comprehensive insights into TAMs and their subpopulations within TME have been discovered. The phenotypes and functions of TAMs in tumor conditions are determined by transcriptional and epigenetic modulations 20, 21, which are greatly influenced by cytokines and metabolites released by cancer cells 22. Understanding the diversity and contribution of TAMs to pathophysiological processes may provide new therapeutic targets for human cancers. Indeed, certain strategies designed to target TAMs have gained remarkable success in pre-clinic studies. However, the effectiveness of these strategies has been limited in clinical trials, highlighting that more precise mechanism and ingenious technologies should be further exploited in this field. In this review, we summarize the recent advancements in TAM research and aim to gain a comprehensive understanding of their roles in cancer immunity and therapy.

## 2. The origin, polarization and heterogeneity of TAMs

### 2.1 The origin of TAMs

First discovered by Ellie Metchnikoff, macrophages are a type of white blood cell that defends the host against pathogens through a process called phagocytosis and engages in innate and adaptive immunity by interacting with other immune cells 23. It has long been held that macrophages originate from blood monocytes produced from myeloid progenitors in bone marrow (BM) 24. Upon tissue injury, infection or carcinogenesis, these circulating monocytes are rapidly recruited to the corresponding site, where they differentiate into macrophages and accumulate in large amounts 25. However, by lineage tracing and fate mapping technologies, cumulative evidence indicates that macrophages can also derive from embryonic progenitors originating from yolk sac or fetal liver, representing another major developmental path of macrophages in addition to monocyte differentiation 26, 27. These embryonic-derived macrophages reside in organs (such as the brain, liver, and skin), proliferate, and maintain locally as TRMs throughout life, referring TRMs either in the liver as Kupffer cells or in the brain as microglia. TRMs can be classified into three subsets based on common life cycle properties and core gene signatures (*Timd4*, *Lyve1*, *Folr2*, and *Ccr2*) in most murine tissues: TLF^+^ macrophages (expressing TIM4 and/or LYVE1 and/or FOLR2), CCR2^+^ macrophages (TIM4^-^LYVE1^-^FOLR2^-^) and MHC-II^hi^ macrophages (TIM4^-^LYVE1^-^FOLR2^-^CCR2^-^). TLF^+^ macrophages are maintained through self-renewal with minimal monocyte input, while CCR2^+^ macrophages are almost entirely replaced by monocytes. MHC-II^hi^ macrophages, on the other hand, receive modest monocyte contribution, but are not continually replaced 27. No matter what the origins are, colony stimulating factor 1 receptor (CSF1R) and its two ligands CSF1 and interleukin (IL)-34 are essential for the differentiation and expansion of macrophages 28. Overall, macrophages are present in almost all tissues and exhibit complex phenotypic heterogeneity and functional diversity under various physiological and pathological conditions because of different developmental origins and tissues of residence.

**Figure 1:**
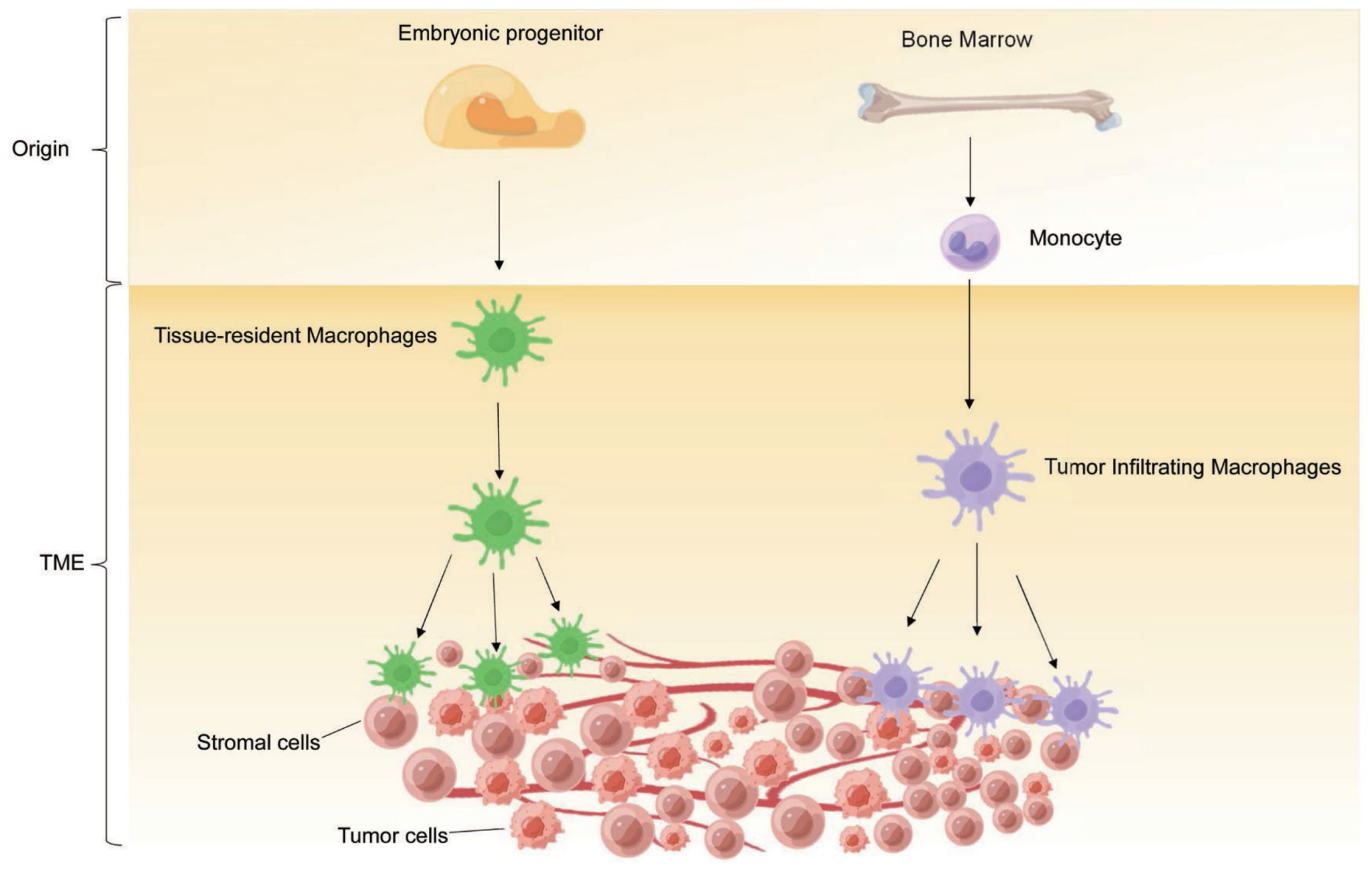
The origin of TAMs. TAMs derive from two main sources: tissue-resident macrophages and newly recruited monocyte-derived macrophages.

In TME, infiltrated TAMs are also composed of both BM-derived macrophages and TRMs (Figure 1). Cancer cells can induce emergency myelopoiesis and expansion of bone marrow myeloid progenitors resulting in increased classical Ly6C^+^ monocytes 29. BM-derived circulating peripheral monocytes are recruited into TME by cytokines and chemokines, such as CSF1, GM-CSF, IL-1β, SDF1α, VEGF and CCL2, and subsequently differentiate into TAMs 30-33. In many cancers, these monocyte-derived macrophages are the main source of TAMs. For example, in a transgenic model of murine breast cancer, TAMs differentiated from monocytes are phenotypically distinct from the predominant mammary tissue macrophages in healthy mammary gland. Monocyte-derived TAMs gradually replace mammary tissue macrophages and promote tumor growth 15. Additionally, retinoic acid, a metabolite of vitamin A1 produced by murine sarcoma tumor cells, selectively suppresses the DC-promoting transcription factor interferon regulatory factor-4 (IRF4) and drives intra-tumoral monocyte differentiation toward TAMs and away from DCs 34.

Meanwhile, the importance of TRMs in sustaining TAM levels and promoting tumor growth in certain types of cancers has been demonstrated by recent studies 17, 26, 35, 36. TRMs are involved in defense, homeostasis, tissue integrity, and wound healing in healthy tissues. Although both embryonic-derived TRMs and monocyte-derived macrophages contribute to the accumulation of TAMs, it is not fully understood which TAMs population functions in regulating tumor progression. For instance, in a mouse model of breast cancer, depletion of TRMs did not reduce the tumor size, whereas depletion of circulating macrophages significantly decreased the tumor volume 15. On the contrary, ablation of BM-derived macrophages did not disrupt tumor progression in a mouse model of pancreatic cancer, but depletion of TRMs dramatically reversed the trend 26. Furthermore, in human breast cancer, FOLR2^+^ mammary resident macrophages in tumors, which are localized in perivascular areas in the tumor stroma, can efficiently prime effector CD8^+^ T cells and are correlated with patient survival 37.

It is noteworthy that TAM populations originating from different sources exhibit distinct temporal and spatial distribution in the TME. In the lung cancer model, macrophages from both origins were found to facilitate tumor growth and progression 38. Moreover, at the early stage of non-small cell lung carcinoma (NSCLC), TRMs accumulated in close proximity to tumor cells and induced potent suppression of adaptive immunity mediated by regulatory T cell 36. During tumor growth, TRMs undergo redistribution towards the periphery of the TME, which becomes dominated by monocyte-derived macrophages in both mouse and human NSCLC. This suggests that TRMs create a pro-tumorigenic niche for early NSCLC cells 36. Nevertheless, these findings support the function complexity and diversity of TAMs, and further studies are needed to address the conundrum.

### 2.2 The polarization of TAMs

It's widely recognized that macrophages are highly plastic cells capable of undergoing specific polarization in different tissue environments. In response to different environmental signals, undifferentiated M0 macrophages which represent the unpolarized and resting state, can be polarized into two types: classically activated macrophages (M1) and alternatively activated macrophages (M2) 39. M1 macrophages, triggered by interferon (IFN)-γ and bacterial lipopolysaccharide (LPS), exhibit increased levels of nitric oxide synthase (NOS) and reactive oxygen species (ROS). These M1 macrophages are considered as anti-tumor cells with secretion of inflammatory factors including IL-6, IL-1, and tumor necrosis factor-α (TNF-α), and promote adaptive immune response by highly expressing antigen presenting MHC complex 40. By contrast, the M2 macrophages, polarized by IL-4, IL-13, and transforming growth factor β (TGF-β), are associated with the initiation, progression, metastasis, and immune evasion of tumors, by secreting anti-inflammatory cytokines such as IL-10, IL-4, and IL-13 41. Moreover, M2 macrophages are much more complex than M1, which can be further classified into M2a, M2b, M2c, and M2-like macrophages (Table 1) 42.

Compared to the classic dual classification of macrophages, TAMs display greater phenotypic and functional diversity. In many cases, TAMs are considered as M2-like macrophages due to their similarities to M2 macrophage properties, such as high expression of ARG1, VEGF, CD206, CD204, and low expression of MHC-II 43. The polarization of TAMs into M2-like phenotype can be induced by tumor-derived lactic acid, mediated by hypoxia-inducible factor 1α (HIF-1α) 44. In addition, the high acidification of the TME caused by lactic acid accumulation, leads to the G protein-coupled receptor (GPCR)-dependent expression of the transcriptional repressor ICER in TAMs, promoting polarization of TAMs towards an M2-like phenotype and facilitating tumor growth 45. However, studies also provide evidence suggesting that TAMs are a mixed population of cells expressing both M1 and M2 markers 46-48. In the early stage of human lung cancer, a mixture of classical tissue monocytes and TAMs was observed with co-expression of M1/M2 markers, as well as T cell coinhibitory and costimulatory receptors 49. These results indicate the complexity of TAMs and the limitation of classic M1/M2 classification.

Advances in single cell omics and mass cytometry by time-of-flight (CyTOF) technologies have provided new approaches to analyze TAM states in more detail. scRNA-seq studies have been conducted in various cancers, including breast cancer, NSCLC, small-cell lung cancer, hepatocellular carcinoma (HCC), glioblastoma, colorectal cancer (CRC), renal cell carcinoma (RCC), and pan-cancer analysis 50-57. These single cell studies have dissected TAMs into multiple distinct clusters based on transcriptomic profiles, which may have different functions in tumor progression. For example, *MMP12*-expressing TAMs in NSCLC, which do not resemble either M1 or M2 cells, are most strongly associated with a poor clinical outcome 58; a high abundance of secreted phosphoprotein 1 (*SPP1*)-expressing TAMs is correlated with worse outcome in NSCLC, CRC, and pancreatic ductal adenocarcinoma (PDAC) 58; inhibiting APOC1 promotes transformation of M2 macrophages into M1 phenotypic macrophage through the ferroptosis pathway, which reshapes the TME and improves anti-PD1 immunotherapy in HCC patients 59; integrated analysis of bulk RNA and single-cell RNA sequencing databases reveals Complete Component 1q (C1Q) ^+^ TAMs as one major anti-tumor immune cell population in osteosarcoma patients 60. In addition, macrophage subsets are found to show heterogeneous transcriptomic patterns among distinct tumor types with several tumor-enriched macrophage subsets were found: the ISG15^+^ TAMs upregulated multiple interferon-inducible genes, the SPP1^+^ TAMs and C1QC^+^ TAMs resembled dichotomous functional phenotypes of TAMs in CRC, LYVE1^+^ macrophages and NLRP3^+^ macrophages were preferentially enriched in non-cancer tissues and likely represented as pro-inflammatory TRMs clusters 21. Similar to previous studies, a single-cell trajectory analysis of macrophages in gastric cancer reveals the existence of two distinct cell states: a proinflammatory "M1-like" state characterized by high CD163 and S100A12 expression, and an "M2-like" state of TAMs with elevated CD163 and FOLR2 expression 61. Further research is needed to identify the phenotypic and functional similarities and the difference between TAM clusters in distinct cancers, in different stages of tumor progression, and in primary and metastatic cancers.

Besides, it is largely unknown how the spatial localization of TAMs within the tumor connects to phenotype and function of TAMs. The development of spatial transcriptomics tools also provides information on spatial distribution information of TAMs, adding a new dimension to our understanding of TAM function in different contexts of cancer. Spatial transcriptomics of TAMs infiltration in NSCLC reveals that TAMs enrichment in the TME is relevant to tumor cell resistance to ICB immunotherapy regardless of its PD-L1 status, which is mediated by CD27, ITGAM, and CCL5 gene expression upregulation within tumor compartment 62. Spatial and single-cell analysis of human normal and cancer colorectal tissues elucidate co-localization of cancer cell with SPP1 ^+^ TAMs at the invasive front of tumor, where CRC cell secrets human leukocyte antigen G (HLA-G) to transform TAMs into macrophages with immunosuppressive feature and reduces cytotoxicity of ICB immunotherapy 63. Likewise, the progress of these cutting-edge technologies will bring new insights and guide the research on the new cancer therapy methods by targeting the unique population of TAMs.

### 2.3 The heterogeneity of TAMs

**Figure 2:**
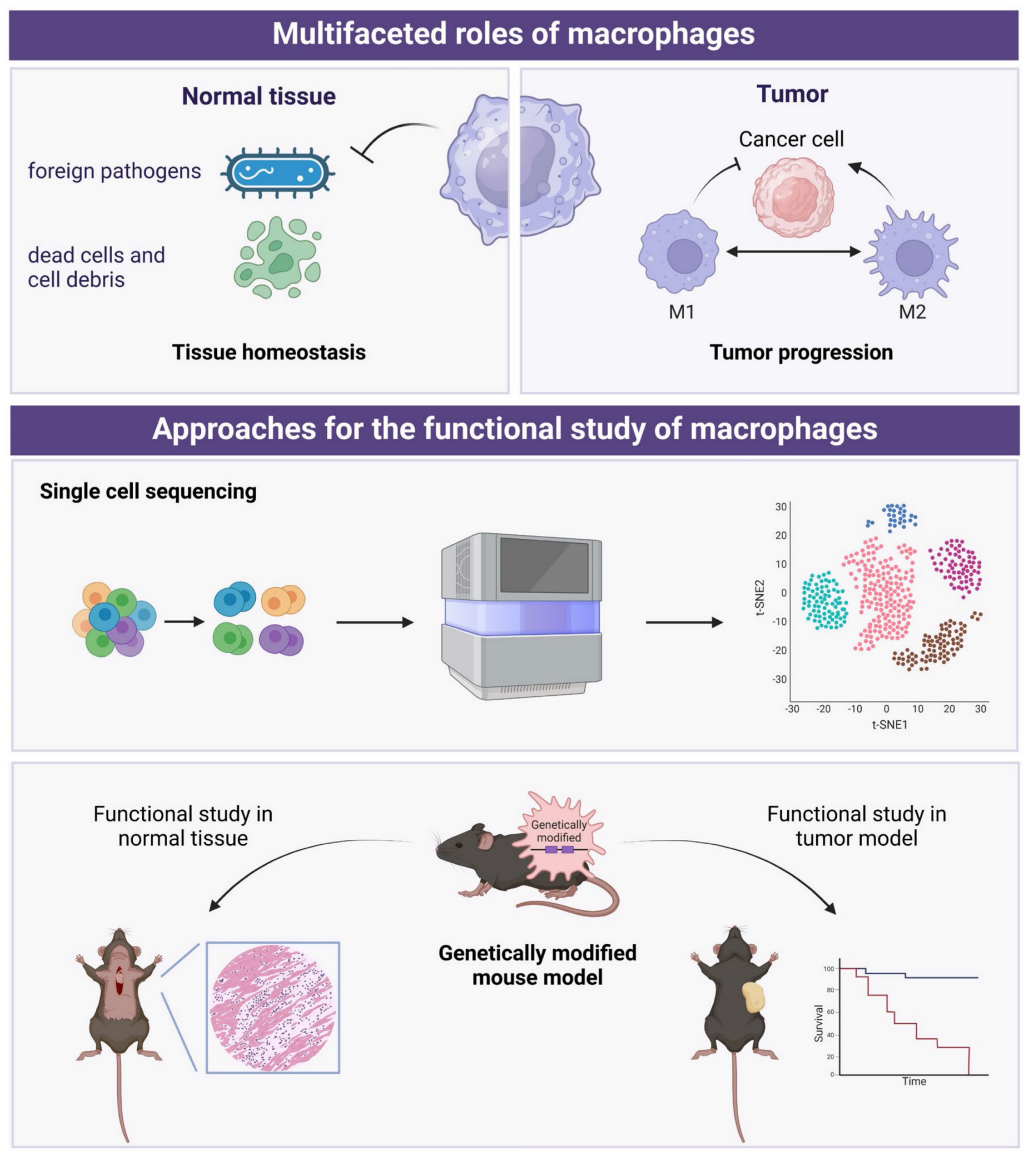
The multifaceted roles of macrophages and the approaches for functional study of macrophages. *Created in BioRender. Zhao, H. (2025) https://BioRender.com/t36o292*.

Due to the multifaceted roles of macrophages in tissue homeostasis and tumor surveillance, the differentiation, activation, and regulation of macrophages within the microenvironment have become major research focuses. Currently, there are two main strategies that dominate the research on macrophages (Figure 2). The first involves using single-cell sequencing (scRNA-seq), a powerful tool to dissect the tumor heterogeneity 64, to categorize macrophages in normal or tumor tissues and functionally annotate the gene expression within each cluster. Building on this, in-depth functional studies are conducted using macrophage-specific genetically modified mice. This includes techniques like knocking out or knocking in specific genes in macrophages, followed by histological examination and functional analysis. Additionally, tumor transplantation models can be constructed on the basis of genetically modified mice to further investigate the impact of specific gene-regulated macrophage functions on tumor progression.

Recent scRNA-seq studies have shown that the traditional categorization of macrophages into M1 and M2 phenotypes is not as clear-cut as previously thought 65. While M1 macrophages are generally associated with pro-inflammatory responses and M2 macrophages with anti-inflammatory responses, scRNA-seq analyses have revealed a more complex landscape of macrophage subpopulations. In a recent study, an extensive analysis of scRNA-seq data from myeloid cells in 380 samples spanning 15 different cancer types was conducted 21. This analysis integrated newly collected data with eight previously published datasets, providing a comprehensive and expansive view of TAMs. By comparing monocytes and macrophages across multiple cancer types, the study consistently identified two distinct subsets of tumor-infiltrating monocytes (TIMs): CD14^+^ and CD16^+^ TIMs. Additionally, a subset of LYVE1^+^ interstitial macrophages were observed in non-cancerous tissues. Furthermore, the analysis revealed seven distinct clusters of TAMs, each characterized by specific marker gene expression patterns. These TAM clusters included INHBA^+^ TAMs, C1QC^+^ TAMs, ISG15^+^ TAMs, LNRP3^+^ TAMs, LYVE1^+^ TAMs, and SPP1^+^ TAMs. These findings shed light on the heterogeneity of TAM populations across various cancer types and non-cancerous tissues. This comprehensive scRNA-seq analysis provides valuable information for understanding the roles and potential therapeutic targets of TAMs in cancer progression and treatment response. In future studies, combined with single-cell sequencing data, new computational methods, such as unsupervised clustering approaches 66, can be considered to identify potential new subtypes of macrophages.

## 3. Macrophages in carcinogenesis and cancer immunity

**Figure 3:**
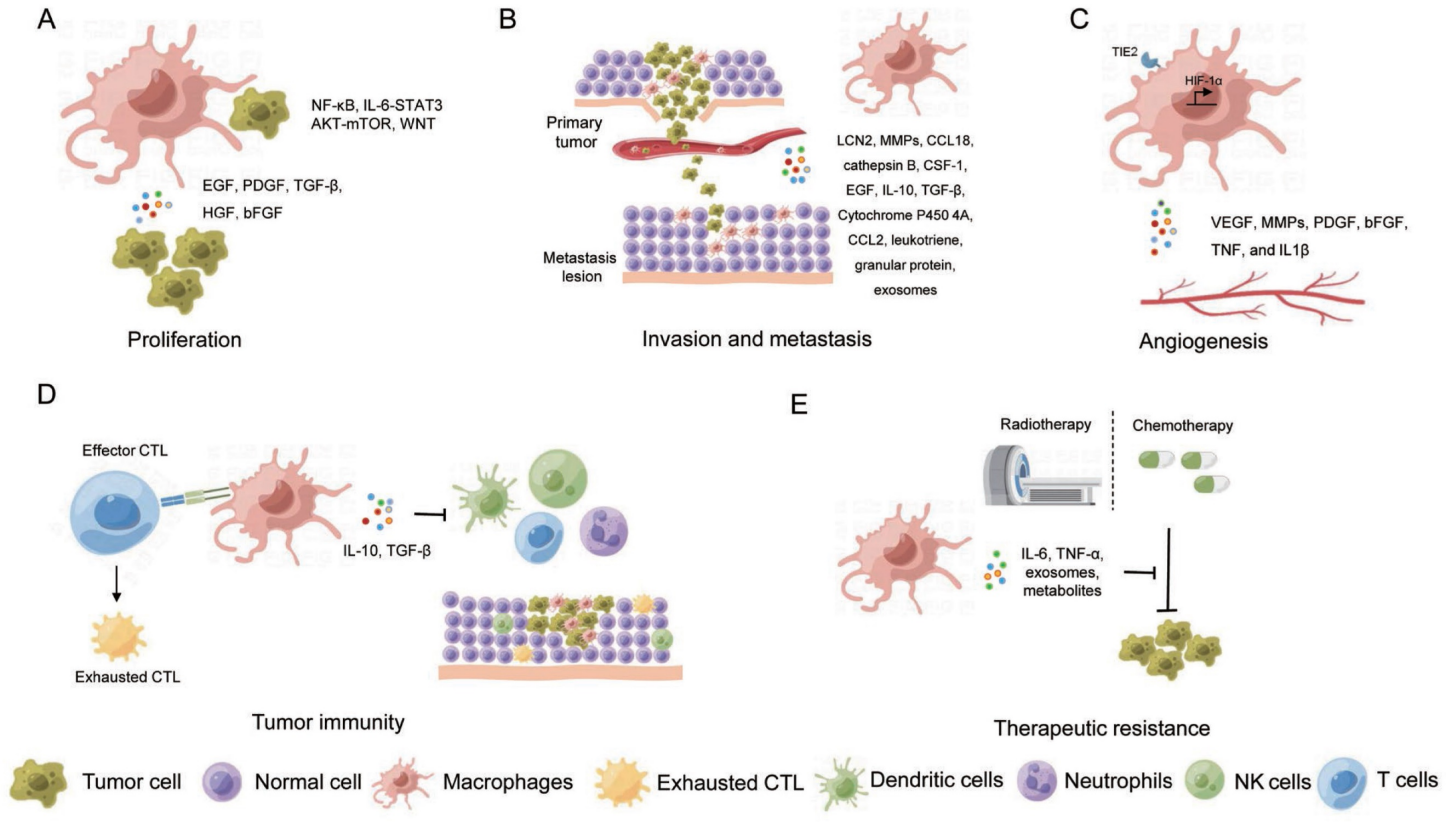
The role of macrophages in cancer development and therapy. (A) Proliferation; (B) Invasion and metastasis; (C) Angiogenesis; (D) Tumor immunity; (E) Therapeutic resistance.

Macrophages exert dual effects in carcinogenesis, with some promoting while others suppressing tumor growth 67, 68. M1-like macrophages execute anti-tumor function by killing the tumor cells through cytotoxic activity directly, attacking cancer cells by cooperation with T cells through antigen present, or secreting cytokines to suppress tumor growth. However, most TAMs promote tumor growth and metastasis by secreting various factors and interacting with other cells in TME, leading to poor prognosis in multiple cancers including breast, cervix, bladder, brain, and prostate cancer 69-73. Furthermore, TME converts M1-like macrophages to M2-like macrophages, which plays an important role in the development and progression of tumors. As discussed above, given the high plasticity and diversity of TAMs, it is crucial to fully understand the properties and functions of transcriptomic unique and spatial unique TAM clusters in regulating tumor initiation and development. Herein, we discuss the roles of TAMs in tumor cell proliferation, invasion, and metastasis, stimulating angiogenesis, tumor immunoevasion, and therapeutic resistance (Figure 3).

### 3.1 Anti-tumorigenic effects of TAMs

Macrophages are reported as the main phagocytic population within TME. By distinguishing cancer cells from normal cells, M1 type macrophages can directly engulf cancer cells by phagocytosis activity and indirectly eliminate tumor cells by inducing cancer cell death through secreting some molecules including ROS and NO or by activating other immune cells such as T cells and nature killer (NK) cells 74. The potential tumor-suppressive role of TAMs has been studied in various tumor contexts. For instance, high infiltration of CD68^+^ TAMs has been associated with improved survival in colon, gastric, and endometrial cancer patients 75-77. In a mouse model of CRC metastasis, depletion of Kupffer cells (TRMs in the liver) resulted in increased liver metastasis of CRC cells, suggesting an inhibitory function of macrophages in liver metastasis 78. In melanoma, CD169^+^ macrophages have been shown to inhibit tumor growth by blocking the dissemination of tumor-derived extracellular vesicles 79. In the single-cell analysis of TAMs, some M1-like TAM subsets and other newly identified TAM populations are correlated with better prognosis, providing further evidence for the existence of an anti-tumorigenic portion of TAMs within TME 80. However, cancer cells have evolved mechanisms to escape uptake by TAMs with the expression of “don't eat me” signal genes such as CD47 and CD24, which disrupt the phagocytosis, and blocking CD47 or CD24 by antibodies can re-activate the macrophage mediated phagocytosis of tumor cells 81, 82.

Furthermore, M1-like TAMs can induce ferroptosis, an intracellular iron-dependent form of cell death, in cancer cells through various mechanisms. These include the release of proinflammatory cytokines, providing peroxides to trigger Fenton reactions, and activating CD8+ CTLs, with the latter being considered a major contributor to initiating ferroptosis in cancer cells 83, 84. The activated CD8+ CTLs produce IFN-γ, which activates JAK/STAT1 pathway and downregulates the transcription of SLC3A2 and SLC7A11, two subunits of the glutamate-cysteine antiporter system x_c_^-^ that involved in ferroptosis 85. This action disables the GSH-dependent antioxidant system and consequently promotes tumor cell excessive lipid peroxidation and ferroptosis 85. Additionally, during the respiratory burst, M1-like TAMs can release peroxides (H_2_O_2_) to trigger intracellular Fenton reaction and generate excessive ROS, therefore promoting tumor cell ferroptosis 86, 87. Interestingly, ferroptosis products of dying cancer cell contrarily promotes TAMs switch into an M2-like pro-tumor phenotype via STAT3-dependent fatty acid oxidation and accelerates pancreatic adenocarcinomas 88, which suggests the crafty characteristics of tumors and the complicated crosstalk between TAMs and cancer cells.

Emerging evidence from scRNA-seq studies has shed light on the discovery of novel macrophage subtypes exhibiting remarkable potential in antitumor activities. One notable investigation found that the presence of CD74^+^ macrophages in hepatocellular carcinomas was strongly associated with improved prognosis and activation of immune response pathways 89. Another study made a significant observation uncovering the role of LC3-associated phagocytosis, a distinct process from conventional autophagy, in driving TAMs to exert control over tumor growth 90. This unique mechanism relies on the participation of tumor-infiltrating T cells and is dependent on the coordinated activation of stimulator of interferon response CGAMP Interactor 1 (STING) and type I interferon responses. In the context of breast cancer, single-cell studies have revealed the presence of a distinct population of folate receptor 2^+^ (FOLR2^+^) macrophages residing in the perivascular regions of the tumor stroma 91. These macrophages engage in interactions with CD8^+^ T cells and demonstrate a remarkable ability to efficiently prime effector CD8^+^ T cells. Notably, a higher density of FOLR2^+^ macrophages within tumors is associated with improved patient survival, highlighting their potential as prognostic markers and their role in facilitating anti-tumor immune responses. In addition to these findings, recent research has highlighted the potential of targeting monoamine oxidase A (MAO-A) to modulate the polarization of TAMs 92. MAO-A, an enzyme located in the mitochondrial membrane, has emerged as a promising therapeutic target due to its involvement in TAM function. Notably, compelling results have been observed in a preclinical study utilizing the B16 melanoma mouse model, in which the pharmacological inhibition of MAO-A enzymatic activity with commercially available inhibitors, commonly prescribed for neurological disorders, demonstrated significant efficacy. This inhibition of MAO-A activity resulted in a remarkable reduction in regulatory TAMs (Reg-TAMs) and a concomitant expansion of TAM subsets characterized by a proinflammatory signature.

### 3.2 TAMs promote carcinogenesis

Rather than exerting an anti-tumorigenic function, TAMs are broadly involved in tumor progression. TAMs collaborate with other immune cells and stromal cells, collectively constructing a special microenvironment for cancerous growth. Meanwhile, TAMs foster cancer progression by interacting with TME or by secreting growth factors such as epithelial growth factor (EGF), platelet-derived growth factor (PDGF), TGF-β, hepatocyte growth factor (HGF), basic fibroblast growth factor (bFGF) that stimulate tumor proliferation 93. For example, in HCC, TAMs induced liver inflammation and subsequent carcinogenesis by releasing IL-6, IL-1β, TNF, HGF, CCL2, and other factors 94. In human endometrial carcinoma, chemokine (C-X-C motif) ligand 8 (CXCL8) secreted by TAMs promoted tumor progression by suppressing the expression of estrogen receptors via homeobox B13 (HOXB13) 95. In PDAC, IL-1β released by TAMs suppressed the expression of 15-hydroxyprostaglandin dehydrogenase (15-PGDH), an enzyme inversely associated with tumor advancement, presence of lymph node metastasis and nerve invasion, and poor prognosis of patients 96. Increased colony-stimulating factors (CSFs) produced by TAMs has also been observed to be related to cancer development across a range of malignancies, including liver cancer, breast cancer, RCC, Hodgkin lymphoma, and ovarian cancer 97.

Furthermore, interactions between cancer stem cells and TAMs are reported to promote tumorigenesis as well 98. For example, TAMs promoted stem cell-like properties of cancer cells by activating the nuclear transcription factor-κB (NFκB) pathway in colon cancer and breast cancer, the IL-6-STAT3 (Signal transducer and activator of transcription 3) pathway in HCC and the AKT-mTOR pathway in RCC 99. Even in 3D engineered microenvironments, TAMs intensified the stem-like properties and malignant phenotypes of ovarian cancer cells through the WNT pathway 100.

In addition to the effects of TAMs on cancer cells, the latter reciprocally polarize TAMs towards states in favor of tumor progression by producing cytokines, chemokines, and metabolites. For instance, substances such as succinate, histamine, CSF1, E3 ubiquitin protein ligase COP1, and β-glucosylceramide released by cancer cells can modulate metabolic state and induce ER stress of TAMs, thereby escalating the generation of pro-tumor TAMs 58, 101-105. In glioblastoma, periostin secreted by tumor stem cells recruited monocyte-derived macrophages from peripheral blood and polarized them into M2-like TAMs to promote tumorigenesis 106. Overall, the mechanisms underlying TAMs promoting tumorigenesis are extremely diverse and display context dependency.

In the context of HCC, a specific subset of M2 macrophages characterized by high expression of C-C motif chemokine ligand 18 (CCL18) and the transcription factor CAMP responsive element modulator (CREM) has been identified through scRNA-seq 107. These M2 macrophages are believed to play pivotal roles in tumor progression. Understanding the association between M2 macrophages, CCL18 expression, and CREM provides valuable insights into the underlying mechanisms driving HCC development and paves the way for targeted interventions to combat this aggressive cancer. In another notable study, a tumor-specific macrophage subpopulation marked by the upregulation of triggering receptor expressed on myeloid cells 2 (TREM2)/apolipoprotein E (APOE)/complement C1q (C1Q) markers has been discovered and validated using advanced imaging techniques 108. This subset of macrophages demonstrated a significant enrichment in tumors from patients who experienced recurrence following surgery, specifically in clear cell renal cell carcinoma (ccRCC). The identification of these TREM2/APOE/C1Q-positive macrophages not only offers a potential prognostic biomarker for ccRCC recurrence but also presents a promising target for therapeutic strategies aimed at preventing tumor relapse. Collectively, these studies shed light on the diverse, context-dependent roles of macrophages in the tumor microenvironment and their considerable impact on cancer progression.

### 3.3 TAMs enhance tumor metastasis

In the theatre of oncology, studies have revealed the critical roles of TAMs in stimulating tumor invasion and metastasis. TAMs can orchestrate a hostile breakout, releasing an arsenal of weapons including matrix metalloproteinases (MMPs), cathepsin, urokinase, protease, and matrix remodeling enzymes. These molecular saboteurs disrupt cell-cell and cell-matrix junction, enabling the escape and invasion of cancer cells 109-113. The plot thickens when sphingosine-1-phosphate (S1P), released by apoptotic tumor cells, stimulated TAMs to secrete lipocalin-2 (LCN2), further propelling tumor metastasis 114. Similarly, in a RCC model undergoing IL-2/anti-CD40 immunotherapy, macrophage-dependent NO in the tumor microenvironment was essential to regulate the activity of MMPs and the expression of adhesion molecules, which was the basis for metastasis 115. TAMs are also capable of igniting cancer cell invasions in other ways. TAMs-derived CCL18 activated the interaction between integrin and receptor membrane-associated phosphatidylinositol transfer protein 3 (PITPNM3) to promote the metastasis of breast cancer 116. TAM-produced cathepsin B has also been shown to enhance breast cancer cell invasion in a lung-metastasis model. The consumption of glucose fuels enhances hexosamine biosynthetic pathway (HBP) and O-GlcNAcylation of cathepsin B in TAMs, which supported cancer metastasis 117. The positive feedback between tumor cells and TAMs triggered tumor cells to secrete CSF-1, stimulating TAMs to secrete epidermal growth factor (EGF), which also accelerated tumor invasion and metastasis by destroying the matrix 118-120. Additionally, TAMs have a hand in regulating epithelial-mesenchymal transformation (EMT), a well-known bioprocess intrinsically linked with tumor metastasis. In pancreatic cancer, for example, TAMs are revealed to bolster EMT and foster cancer metastasis by reducing the expression of E-cadherin via activating the TLR4/IL-10 signaling pathway 121, 122. In another study, TAMs were demonstrated to promote EMT and metastasis of liver cancer and CRC through secreting TGF-β 123.

In addition to the assistance in metastasis at primary sites, studies have shown that TAMs play a crucial role in preparing a favorable landing strip for migratory cancer cells, assisting these rogue cells to seed in distal tissues or organs. For instance, cytochrome P450 4A released by TAMs, fostered the formation of pre-metastasis niche and the trend of M1 polarization 124. In lung metastasis models of breast cancer and melanoma, monocytes were recruited by CCL2 produced by cancer cells to differentiate into macrophages, creating a pre-metastatic niche for tumor cells 125, 126. In a liver metastasis model of PDAC, TAMs derived inflammatory monocytes, are able to secrete granular protein to transform the resident hepatic stellate cells into myofibroblasts to support cancer cell implantation 127. Interestingly, TRMs can also facilitate cancer metastasis into the tissue, due to their anti-inflammatory property. For example, Alveolar macrophages, TRMs in the lung, promoted lung metastasis of HCC and breast cancer by secreting leukotriene and suppressing the Th1 response 128. Peritoneal TRMs promoted ovarian cancer metastasis into the peritoneal cavity by driving the spheroid formation 129. Besides, TAMs are affected by exosomes produced by tumor cells, for example, exosome-educated macrophages boosted liver metastasis of pancreatic cancer through TGF-β secretion 130.

Recent advances in single-cell research have provided invaluable insights into tumor microenvironment modulation and the intricate relationship between macrophages and cancer cell invasion. These studies not only highlight the impact of macrophages on cancer cell behavior but also spotlight their potential as therapeutic targets. For instance, a lung cancer study revealed that the depletion of tissue-resident macrophages led to significant changes in the tumor microenvironment, curbing tumor invasiveness and growth 36. These alterations included a decrease in regulatory T cell numbers and a shift in their phenotype, accompanied by an accumulation of CD8^+^ T cells. Furthermore, the relocation of tissue-resident macrophages from the tumor core to the periphery during tumor progression indicated their dynamic role in lung cancer development. Another study focused on glioblastoma demonstrated the ability of macrophages to induce a transition of glioblastoma cells into mesenchymal-like states 131. This transition was mediated by the secretion of oncostatin M by macrophages, which interacted with its receptors on glioblastoma cells, activating the signal transducer and activator of transcription 3 (STAT3) signaling pathway. Importantly, the acquisition of mesenchymal-like states in glioblastoma cells was associated with increased expression of a mesenchymal program in macrophages and enhanced cytotoxicity of T cells. These findings highlight the extensive alterations in the immune microenvironment orchestrated by macrophages and underscore their potential therapeutic implications.

### 3.4 TAMs enhance angiogenesis

In solid tumors, TME is typically characterized by a state of hypoxia, an essential element for tumor angiogenesis, which is recognized as one of the hallmarks of cancer 132. A wealth of research has evidenced that hypoxic TME steers the recruited macrophages towards an M2-like state, inciting TAMs to unleash pro-angiogenic factors such as vascular endothelial growth factor (VEGF), HIF, MMP, PDGF, bFGF, TNF, and IL-1β 133, 134. For example, in breast and colon cancer, TAMs were positively correlated with VEGF level and microvascular density 135, 136. TAM-induced MMP9 has been found to be a strong ally in promoting tumor angiogenesis in ovarian cancer, while TAM-derived thymine phosphorylase (TP) has been implicated in fostering tumor angiogenesis in gastric cancer 137, 138. Another piece of this intricate puzzle is the role of WNT7b, produced by TAMs, which ratchets up the expression of VEGF-A in vascular endothelial cells, thereby enhancing angiogenesis in breast cancer 139. The significance of HIF expression in TAMs can also not be overstated for tumor angiogenesis, as supported by the observation that knockout of HIF-1α in TAMs resulted in curtailed angiogenesis and a reduction in tumor burden in breast cancer. Additionally, TIE2-expressing monocytes, a particular breed of TAM existed both in human peripheral blood and tumors, has been noted to fuel tumor angiogenesis and tumor growth in endometriosis lesions, pancreatic cancer, and ovarian cancer 140, 141.

### 3.5 TAMs in tumor immunity

In addition to their direct effects on cancer cells, TAMs function as pro-tumorigenic cells by attenuating cancer immunity to construct an immunosuppressive microenvironment for cancer cell growth in several ways 142, 143. As phagocytes, TAMs compete with dendritic cells and degrade tumor-associated antigens (TAA) in TME 144. Meanwhile, antigen presentation activity is abnormal in TAMs, resulting in inhibition of adaptive immune response and thereby facilitating tumor immune evasion. This is evident where the transcription factor IRF8 is required for cancer cell antigen presentation by monocyte-derived TAMs, which was essential for promoting cytotoxic T lymphocyte (CTL) exhaustion within the tumor. Notably, TAM-specific IRF8 deletion prevented exhaustion of cancer cell-reactive CTLs and suppressed tumor growth 145.

TAMs also impede the anti-tumor activity of tumor-infiltrating natural killer cells and T cells and synergize with myeloid-derived suppressor cells (MDSCs), tumor-associated dendritic cells, and neutrophils to foster an immunosuppressive tumor microenvironment 142, 144. T cell immunity is suppressed by TAMs as evidenced by the unleashed T cell response upon TAM blockage in several cancers 146, 147. Recruitment of CD8^+^ T cells was blocked by TAMs in TME of HCC through the inhibition of CXCL9 and CXCL10, meanwhile CD8^+^ T cell activation and proliferation were attenuated through regulating L-arginine by ARG1 and inducible NO synthase (iNOS) in lung cancer and lymphoma 44, 148. TAM-secreted cytokines including IL-10 and TGF-β, inhibited T cell proliferation and differentiation and promoted T cell exhaustion 149, 150.

Furthermore, TAMs contribute to the blockade of cytotoxic activities in T cells, natural killer T cells, and natural killer cells on account of the high expression of immune checkpoint ligands on TAMs such as programmed cell death protein ligand 1 (PD-L1), programmed cell death protein 1 (PD-1), B7-H4, and cytotoxic T-lymphocyte-associated protein 4 (CTLA4), which leads to reinforced tumor growth 147, 151-153. For example, in mouse models of colon cancer and breast cancer, M2-like TAMs expressed high levels of PD-1, which not only reduced the anti-tumor function of T cells but also inhibited the phagocytosis of macrophages and promoted the growth of tumors 154. Additionally, a wealth of data reveals that TAMs can also mediate T cell depletion in TME. The activated antigen-specific Fas^+^CD8^+^ T cells undergo apoptosis following their interaction with FasL^+^CD11b^+^F4/80^+^ monocyte-derived macrophages within the liver, which systemically depleted the peripheral T cell numbers and diminished tumoral T cell diversity and function by siphoning activated CD8^+^ T cells from circulation 18. Importantly, TAMs and M-MDSCs, but not cancer cells, consumed the most glucose per cell in TME and maintain robust glucose metabolism 155, implying that TAMs could trigger T cell death by glucose deprivation and lactate production 156. Taken together, all these results highlight macrophage as a central player of the immunosuppressive TME through regulating the recruitment and the function of multiple immune subtypes.

### 3.6 TAMs in therapeutic resistance

Growing studies have indicated the significant roles of TAMs in chemo- or radio-resistance. Generally, TAMs contribute to therapeutic resistance either by promoting cell survival and cancer cell stemness or by shielding cancer cells from death. For example, TAMs have been reported to activate the STAT3 signaling pathway in cancer cells by producing cytokines such as IL-6 and TNF-α, which enhanced the resistance to chemotherapy in various cancer cells 157, 158. It was also shown that TAMs can secret exosomes containing microRNAs and metabolites that are implicated in chemotherapy resistance in different type of cancers including ovarian cancer, gastric cancer, and PDAC 130, 159, 160. In addition, blockage of TAMs or certain factors secreted by TAMs has shown to improve the radiotherapy sensitivity in head and neck cancer as well as breast cancer 161, 162.

## 4. TAM-targeted cancer therapy

**Figure 4:**
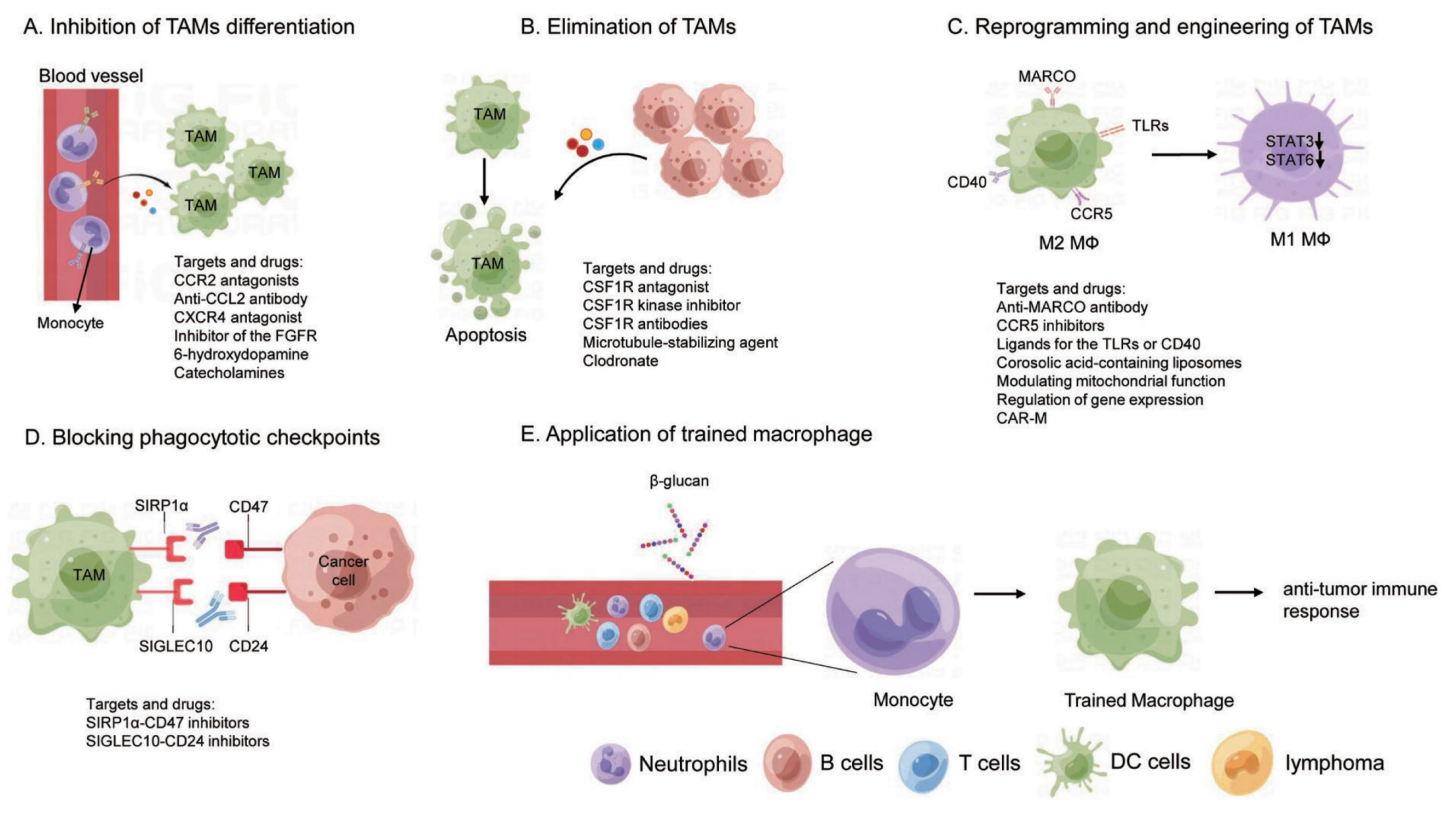
TAM-targeted cancer therapy. (A) Inhibition of TAMs differentiation; (B) Elimination of TAMs; (C) Reprogramming and engineering of TAMs; (D) Blocking phagocytotic checkpoints; (E) Application of trained macrophage.

Conventional cancer treatments, including surgical resection and kinase inhibitors, frequently encounter challenges such as tumor relapses and drug resistance 163, 164. This underscores the urgent need to develop novel therapeutic strategies for more effective cancer therapy. Given the importance of TAMs in tumor progression and immune response, targeting TAMs as a potential cancer therapeutic strategy has aroused great interests. Numerous approaches are either being developed or are under active research for different types of cancer, with many clinical trials currently underway. These strategies are designed either through inhibiting the pro-tumorigenic function or boosting the anti-tumorigenic capabilities of TAMs by manipulating the mass, the states, and the activity of TAMs. This discussion will center on the current macrophage-targeting therapies, that can be broadly divided as follows: inhibition of monocyte/macrophage recruitment, depletion of macrophages, reprogramming and engineering of TAMs, and other therapies (Figure 4).

### 4.1 Inhibition of monocyte/macrophage recruitment

The strategy of inhibiting the recruitment of monocyte into tumor tissue holds promise, as TAMs are predominantly derived from circulating monocyte precursors. Chemokine ligand 2 (CCL2) is essential for the recruitment and localization of monocytes into tumors 165, making the targeting CCL2 and its receptors CCR2 a viable method for curtailing monocyte infiltration and TAM production. In preclinical models, blocking CCL2-CCR2 signaling by genetic approach or small molecular inhibitors resulted in reduced tumor growth and metastasis and improved the efficacies of chemotherapy, radiation therapy, and immunotherapy in HCC 166-168. In a mouse model of pancreatic cancer, CCR2 antagonists blocked the mobilization of CCR2-positive monocytes from bone marrow into tumors, thereby limiting TAM production and curbing tumor growth and metastasis 169. Carlumab, an anti-CCL2 monoclonal antibody, has demonstrated promising results in preventing the development of certain cancers in mouse models 170. Several small molecular inhibitors and antibodies aimed to disrupt the CCL2-CCR2 axis are under clinical trial. The CXCL12-CXCR4 pathway is another potential target to decrease TAM recruitment, the blockade of which mobilized CD8^+^ T cells to the tumor and reduced TAM accumulation in multiple cancers 171-173. Meanwhile, a peptide antagonist of CXCR4, named as motixafortide, is currently under teste in ongoing clinical trials 174.

Other molecules such MAC-1 (CD11b/CD18) and fibroblast growth factor receptor (FGFR) have also been reported as potential targets. For example, inhibition of MAC-1 has been shown to enhance tumor response to radiation therapy by reducing myeloid cell recruitment, consequently attenuating squamous cell carcinoma growth 175. Likewise, AZD4547, an inhibitor of the FGFR tyrosine kinase family, has been observed to block the FGFR in a lung adenocarcinoma mouse model, resulting in robust TAM elimination and tumor regression, rendering this receptor a potential therapeutic target 176. The potential of targeting 6-hydroxydopamine catecholamines, CSF-1R, and CD88 for cancer therapy in lung cancer and colon cancer as well 28, 177, 178.

### 4.2 Reduction and clearance of TAM

#### 4.2.1 Inhibition of TAMs differentiation

As discussed above, CSF1R is the key factor for TAM survival and proliferation and is highly expressed across all TAM states. This makes the interruption of the CSF1-CSF1R axis a promising method to reduce TAMs.

Firstly, the inhibition of CSF1-CSF1R signaling has resulted in substantial cell apoptosis of TAMs and improvement in T cell response in many tumor models 179-181. The small molecule CSF1R antagonist named PLX3397 (Pexidartinib), has been found to penetrate the blood-brain barrier and significantly reduce the amount of tumor-associated microglia, thereby preventing tumor invasion in a glioblastoma mouse model 182. In murine breast-to-brain metastasis models, the combination of BLZ945, an inhibitor of CSF1R, and AC4-130, an inhibitor of CSF2Rb-STAT5 signaling, has proven effective in controlling tumor growth, normalizing of microglia activation states, and mitigating neuronal damage 183. For advanced ovarian cancer patients, GW2580, a CSF1R kinase inhibitor, has been reported to inhibit macrophage function, reduce M2 macrophage infiltration, and significantly decrease the number of ascites 184. In addition to compounds, CSF-1R antibodies (such as Emactuzumab) are also developed to block the CSF1-CSF1R pathway and have proved its efficacy in diffuse-type giant cancer cells 185, 186. Secondly, the efficacy of chemotherapy or ICB has been found to be improved when applied to block the CSF1-CSF1R axis. For example, docetaxel (microtubule-stabilizing agent) coupled with anti-CSF1R led to TAM depletion in a murine epithelial ovarian cancer model 187. Finally, many ongoing CSF1-CSF1R targeting trials are evaluating their anti-tumor efficacy either alone or in combination with other drugs such as chemotherapy agents or immune checkpoint inhibitors.

### 4.2.2 Elimination of TAMs

Macrophages always undergo transcriptionally and epigenetically remodeling to adapt to the local microenvironment. Targeting the intrinsic regulators of TAMs provides a specific way to deplete the tissue specific TAMs without defects induced by general depletion of monocytes/macrophages. In peritoneal cavity, for example, transcription factor GATA6 is critical for the peritoneal macrophage differentiation and maintenance 188, 189. The depletion of GATA6 in peritoneal TRMs induces cell apoptosis and number loss, indicating that targeting GATA6 can be used to eliminate peritoneal TAMs. Retinoid X receptors (RXRs) determine the identity of peritoneal TRMs by regulating the chromatin accessibility of GATA6. RXRs deficiency impairs neonatal expansion of the large peritoneal macrophages (LPMs) pool and reduces the survival of adult LPMs through excessive lipid accumulation. Depletion of RXR diminished LPMs accumulation in ovarian cancer and strongly inhibits tumor progression in mice 190.

Novel artificial materials have been developed to eliminate the TAMs as well. For example, trabectedin and lurbinectedin could reverse the immunosuppression effect of TAMs through depleting macrophages in the TME. However, these two chemicals inevitably caused side effects due to unselectively macrophage consumption, potentially disturbing immune homeostasis 191. The clodronate liposome, a non-nitrogen bisphosphonates which elicits toxic effects on macrophages via phagocytosis, has been used to deplete TAMs *in vivo*, resulting in reduced tumor growth in PDAC 192 and ovarian cancer metastasis 16. Depletion of TAMs with clodronate has also been shown to prevent aerobic glycolysis and tumor hypoxia, improving tumor response to chemotherapy 193. Moreover, as a result of TAM depletion, PD-L1 expression, as well as T-cell infiltration, is significantly increased in aerobic cancer cells, which dramatically promoted the antitumor efficacy of PD-L1 antibodies 193. Zoledronate, a third-generation nitrogen-containing bisphosphonate, has been shown to exhibit selective cytotoxicity towards TAMs, impairing differentiation of monocytes into TAMs and to reducing the infiltration of TAMs, which finally resulted in decreased tumor angiogenesis and inhibited tumor progression 194.

Furthermore, therapies using Fc domain enhanced anti-TREM2 monoclonal antibody have been developed to promote anti-tumor immunity by eliminating and modulating TAM populations, which leads to enhanced CD8^+^ TIL infiltration and effector function 195. In addition, chimeric antigen receptor (CAR) T cells, genetically modified to express receptors that recognize TAMs-specific antigens, are designed to eliminate TAMs. In an ovarian cancer study, both mouse and human FRβ-specific CAR T cells recognized and depleted the FRβ^+^ TAMs, interrupting ovarian cancer metastasis 196.

### 4.3 Reprogramming of TAMs

Macrophages demonstrate a high degree of plasticity, enabling them to adapt to variable microenvironments. This adaptability paves the way for the reprogramming of TAMs into a tumoricidal phenotype, thereby restoring their anti-tumor effects 197. The reprogramming of M2-like TAMs into M1-like TAMs within the TME has shown promising results. Several surface markers of TAM can be targeted to switch their phenotypes, such as the scavenger receptor MARCO, toll-like receptors (TLRs), CD40, or CCR5 198-201.

In models of breast and colon carcinoma as well as melanoma, an anti-MARCO monoclonal antibody has been developed and has exhibited anti-tumor effects in some cases through reprogramming TAMs to pro-inflammatory phenotypes and enhancing tumor immune responses 198. Similarly, CCR5 inhibitors such as maraviroc, vicriviroc, TAK-779, and anibamine have shown anti-tumor effects in mouse model of multiple cancers and are tested clinically in breast cancer, colon cancer and PDAC 202. In addition, specific ligands for the TLRs or CD40 have also been identified to activate M1 macrophages. The TLR7 agonist imiquimod has been approved by the FDA for topical treatment of superficial basal cell carcinoma 203. TLR3 agonist poly-ICLC, which activates the NFκB pathway and anti-tumor immunity, is under clinical test for glioma 204. Paclitaxel decreases tumor growth by reprogramming TAMs to an M1 subtype in a TLR4-dependent manner 205. Anti-CD40 antibodies have shown significant anti-tumor activity as single agents in several preclinical models including PDAC and breast cancer 206-208. Combined administration of monophosphoryl lipid A (MPLA) and IFN-γ stimulates type I IFN signaling in breast cancer, which reprogramed CD206^+^ TAMs to iNOS^+^ TAMs, resulting in cytotoxic T cell activation through macrophage-secreted IL-12 and TNF-α, finally reduction of primary tumor growth and metastasis 209.

Specific pathways involving anti-inflammatory responses can also be modified to reshape TAMs. For example, by specifically targeting STAT3 through CD163-targeted corosolic acid-containing liposomes, M1-like TAMs were reprogrammed, resulting in a decrease in IL-10 expression and increase in pro-inflammatory TNF-α 210. Similarly, it has been shown that several synthetic molecules (AS1517499, TMC-264, A771726) inhibited STAT6, one of the major signal transducers activated by IL-13 and involved in M2 polarization, leading to inhibited TAMs transformation and tumor progression in a mouse model of breast cancer 211. Furthermore, inhibiting STAT6 transcriptional activity by enhancing STAT6 acetylation suppresses TAMs M2-like polarization, reshapes TME into a tumor-suppressive state, and represses tumor progression in melanoma 212. PI3Kγ, a key macrophage lipid kinase, selectively drives immunosuppressive transcriptional programming in macrophages which promotes tumor immune invasion 213, 214. PI3Kγ signaling in TAMs inhibits NFκB activation and stimulates CCAAT/enhancer binding protein (C/EBP)-β activation through AKT and mammalian target of rapamycin (mTOR), thereby induces a transcriptional program of immunosuppression 213. Genetic depletion of *Pik3cg* or selective pharmacologic targeting of PI3Kγ by IPI-549 reprogramed TAMs, reshaped the TME, and promoted CTL-mediated tumor regression 213-215.

A few other strategies are studied likewise to manipulate TAMs toward to M1-like states. Modulating macrophage mitochondrial function could be considered as an approach to activating TAMs reprogramming. Under hypoxia condition, nuclear-encoded mitochondrial *pyruvate dehydrogenase beta* gene expression is attenuated by promoting Nuclear Respiratory Factor 1 (NRF1) degradation, dampening hypoxia-mediated NRF1 degradation decreases the Warburg effect and promotes M1 polarization of TAM, promoting tumor cells to become more sensitive to apoptosis through a FADD-dependent manner 216. Depletion of NF-κB effector molecule Gadd45b in myeloid cells recovered the activation of pro-inflammatory TAMs and increased intratumor immune infiltration, thereby diminishing HCC and ovarian cancer oncogenesis in mouse 217. For NSCLC patients, disrupting Angptl2, a secreted inflammatory glycoprotein, may be an effective strategy to re-educate TAM polarization and reprogramming of M2-like TAMs to M1-like TAMs 218.

### 4.4 Blocking phagocytotic checkpoints

The therapeutic exploitation of innate immune clearance of dying cancer cells has emerged as an exciting new area of cancer immunotherapy. Similar to the immune checkpoints on T cells, several phagocytotic checkpoints on macrophages have been identified to modulate the tumor-associated antigens uptake, presentation, and degradation. Targeting these phagocytotic checkpoints is critical for tumor clearance and type I IFN immune response. Some cancer cells express “don't eat me” signal ligands such as CD47 and CD24, which can be recognized by TAM receptors such as SIPR1a (for CD47) and SIGLEC10 (for CD24), effectively blocking the attack from TAMs. Interrupting SIPR1α-CD47 or SIGLEC10-CD24 axis by CD47 or CD24 antibodies stimulated TAMs to phagocytose cancer cells and enhanced antitumor T cell responses in mouse models 81, 219, 220. Furthermore, a phase I trial involving an anti-CD47 antibody Hu5F9-G4 demonstrated partial remissions in two patients with ovarian/fallopian tube cancers for 5.2 and 9.2 months 221. As a general marker of embryonic-derived TRMs 222-224, T cell immunoglobulin and mucin domain-containing molecule-4 (TIM4) mediates the uptake of apoptotic cell by recognizing phosphatidylserine (PS). Interestingly, TIM4^+^ cavity TAMs sequester and impair CD8^+^ T cells proliferation through the recognition between TIM4 and PS, which is elevated on activated T cells. Hence, the TIM4 blockade abrogated this sequestration, restored T cell proliferation, and thus enhanced anti-tumor efficacy in models of anti-PD-1 therapy and adoptive T cell therapy in mice 19. Additionally, TIM4-mediated uptake and degradation of dying tumor cells are important for the immune evasion via the canonical autophagy due to reduced antigen presentation 225. Besides, TIM4 functions with LC3-associated phagocytosis (LAP) to promote immune tolerance and blockage of TIM4 with antibody releases the STING-mediated type I interferon responses in TAMs 90. Consistently, blockade of phagocytic receptor MerTK with antibody also resulted in accumulation of apoptotic cells within tumors and triggered a type I interferon response which stimulated T cell activation and synergized with anti-PD-1 or anti-PD-L1 therapy 226.

### 4.5 Application of trained macrophage

The application of trained immunity in macrophages provides a potential strategy for cancer treatment. Traditionally, innate immunity has been understood to react rapidly and nonspecifically upon encountering a pathogen, without building up immunological memory akin to adaptive immunity. However, studies have shown that prototypical innate immune cells (such as monocytes, macrophages, or natural killer cells) have the potential for increased responsiveness upon secondary stimulation, a phenomenon termed “trained immunity” 227, 228. Contrary to the stringent antigen/pathogen specificity of adaptive immunity, trained innate immune cells can trigger systemically enhanced immune responses to a variety of heterologous stimulants after primary stimulation 228, 229. Capitalizing on this characteristic, trained immunity has been leveraged to disrupt the immunosuppressive TME and boost the systemic anti-tumor response via pre-stimulating the myeloid cells. For example, trained immunity induced by pre-treatment of mice with β-glucan, a fungal-derived prototypical agonist of trained immunity, has been associated with transcriptomic and epigenetic rewiring of granulopoiesis and neutrophil reprogramming toward an anti-tumor phenotype 230. Meanwhile, β-glucan also attracts circulating monocyte/macrophages influx into the pancreas with features of trained immunity to exert anti-tumor functions 231. Furthermore, the metabolite S1P mediated whole β-glucan particle (WGP) induced trained immunity in lung interstitial macrophages, leading to inhibition of tumor metastasis and prolonged survival in multiple mouse models of metastasis. Application of WGP-trained BM-derived macrophages through adoptive transfer reduced tumor lung metastasis 232. Interestingly, a recent study also observed that acute respiratory viral infections induced trained immunity in lung tissue-resident alveolar macrophages. These macrophages are poised to exert long-lasting tissue-specific anti-tumor immune response 233, suggesting that trained immunity in macrophage can provide a reprogrammed and persistent activation of immune response. Consequently, a designed nano-therapy has been developed to specifically induce trained immunity with nanoparticle MTP10-HDL in a B16F10 mouse melanoma model to overcome the immunosuppressive tumor microenvironment and synergize with immune checkpoint inhibitors 234. Therefore, creating and modulating the trained immunity in monocyte/macrophage should enhance the anti-tumor immune responses, which might be a novel and promising immunotherapy against advanced cancer and metastasis.

### 4.6 The potential of engineered CAR-macrophages in cancer therapy

**Figure 5:**
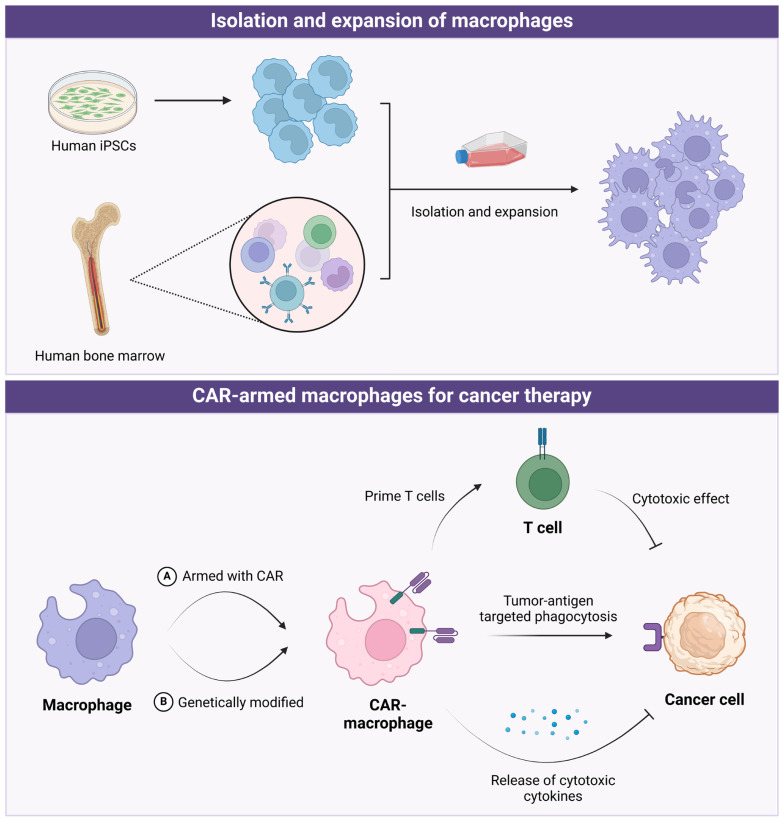
The application of CAR-armed macrophages in cancer therapy. *Created in BioRender. Zhao, H. (2025) https://BioRender.com/x76e687*.

Earlier research focused on macrophage functions and their anti-tumor properties, but recent studies have shifted toward utilizing macrophages directly as therapeutic tools (Figure 5). The laboratory methods to obtain macrophages involve isolating mononuclear cells or monocytes from bone marrow or peripheral blood, and then stimulating, amplifying, and differentiating them *in vitro* (e.g., with GM-CSF and IFN-γ). A recent study used induced pluripotent stem cells (iPSCs) to obtain macrophages after in-vitro differentiation 235. Based on this, macrophages can be further armed with chimeric antigen receptors (CARs), adding a second signal within the macrophages. Similar to CAR-T cells, macrophages armed with CARs offers several benefits: firstly, CAR can precisely target and kill tumors by recognizing tumor-specific antigens on their surface; secondly, it can act as an antigen-presenting cell to prime and activate T cells; and thirdly, further genetic modification of macrophages may enhance their cytokine secretion capabilities, thereby improving their tumor-killing effectiveness.

Based on the ability of macrophages to clear pathogens and antigens, engineered macrophages by modifying antigen receptors on macrophages have also been developed, known as CAR-M (chimeric antigen receptor macrophage) cells. Macrophages engineered with targeted CARs can enhance its antigen presentation and phagocytic capacity, through which CAR-M cells could recognize antigens expressed specifically on cancer cells, therefore attacking and eliminating malignant cells. Zhang Jin's team developed CAR-expressing macrophages using iPSCs as the cell source, referred to as first-generation CD3ζ-based CAR-macrophages (iMACs)^236^. Building on this, they further developed iMACs with toll-like receptor 4 intracellular TIR (Toll/IL-1R) domain-containing CARs and M1 polarization characteristics, which demonstrated enhanced orthogonal phagocytosis, polarization, and superior antitumor functions in treating solid tumors 235. Yizhao Chen and colleagues developed CAR-M targeting HER2 and CD47, demonstrating their inhibitory effects on HER2 or CD47-positive ovarian cancer *in vitro* and *in vivo* 237. The study preliminarily confirmed that these effects are primarily due to phagocytosis, the promotion of adaptive immunity, and modulation of the tumor microenvironment 237. Another recent preclinical study by Zahir Shah and colleagues demonstrated that iPSC-derived CAR-M targeting the tumor antigen PSCA exhibit strong antitumor activity against human pancreatic solid tumors both *in vitro* and *in vivo* 238. Genetically engineered CAR-M targeting HER2 decreased tumor burden in a mouse model 239, 240. Delivery of Adenovirus-delivered CAR to macrophages transforms M2 macrophages into M1 polarization, reshaping TME and amplifying anti-tumor cytotoxicity of T cells, which inhibits lung cancer metastasis during ovarian cancer treatment 240.

The majority of CAR-M strategies are currently in pre-clinical trials, with some already progressing to clinical trials. As an example, the first-in-human multi-center trial utilizing CAR-M carrying an adenoviral vector Ad5f35 targeting HER2 in various HER2-overexpressing solid tumors is currently in Phase I of interventional clinical trials (NCT04660929, estimated completion time: 2024-12), this has demonstrated promising results in effectively targeting solid tumors (https://classic.clinicaltrials.gov/ct2/show/NCT04660929). The phase I clinical trial results of the CAR-M product (CT-0508) demonstrate its preliminary safety, tolerability, and manufacturing feasibility for HER2+ tumors 241. All the above studies elucidate CAR-M is anticipated to emerge as the forefront of tumor immunotherapy.

## 5. Future Prospective

Macrophages are important innate immune cells that play critical roles in clearing pathogens and maintaining tissue homeostasis. As the dominant myeloid cells infiltrate TME, TAMs influence cancer progression and immune response through multiple routes. Co-existence of two distinguished polarizations of TAMs displays spatial and temporal distribution in different types of cancer. M1-like TAMs activate the immune system and suppress tumor progression, whereas M2-like TAMs suppress the immune system to promote tumor development. Cancer cells and other infiltrated cells in TME tend to repress the anti-tumorigenic function and activate the pro-tumorigenic effects of TAMs, which provides a potential approach to take advantage of the M2-like TAMs by switching their polarization to M1-like.

High plasticity is the core characteristic of macrophages, giving rise to phenotypic diversity and functional complexity of TAMs. Although macrophage infiltration is a shared property in different tumors, substantial differences in TAM phenotypes and roles are observed in tumors arising in or disseminating to different tissues. As proof, while TAM infiltration is correlated with poor prognosis in majority tumors, there are noteworthy exceptions such as primary CRC. Advanced technologies have identified increasing subgroups of TAMs and progressively expanded our understanding of TAMs beyond the simple dual classification. This certainly leads to many open questions for future studies. First, functional specificity of unique TAM subsets needs to be elucidated at single cell level, especially in different genetic and tumor contexts. Second, mechanisms underlying TAM regulation on tumor development at primary site and metastatic lesions need more comprehensive analyses since the tissue intrinsic properties vary a lot. Third, spatial distribution of TAMs and their corresponding function within tumors should be explored. Last but not the least, more studies are needed to decipher the master transcriptional and epigenetic regulators accounting for pro- or anti- tumorigenic function of TAMs. These explorations will shed new insights into the fundamental biology of TAM and cancer immunotherapies targeting TAMs.

Given the high infiltration of TAMs in TME, approaches are developed for cancer treatment by depleting macrophages. Despite scientific advancements and promising preclinical studies, the translation of TAM-targeting therapies into effective clinical applications is still challenging. One of the reasons could be the heterogeneous nature of macrophages, which exhibit diverse phenotypes even within the same tumor. Another challenge is related to drug delivery. Many TAM-targeting agents fail to reach the tumor site due to the physiological barriers within the TME. Advanced drug delivery systems, such as nanoparticle-based delivery, are currently being explored to improve drug bioavailability. The side effects of these methods should be evaluated properly since macrophages are widespread and essential for normal tissue homeostasis.

TAMs are mainly replenished by the circulating myeloid precursor pool, which gives rise to the exploitation of cancer therapy by TAM recruitment disruption. One feasible idea is that we could make use of the strong attraction of macrophages to tumor tissues, to engineer T cells to overcome the poor recruitment of T cells at tumor site. This thought requires a profound understanding of the molecular basis of TAM recruitment and may broaden the application of CAR T cells in cancer immunotherapy. While high plasticity makes reprogramming TAMs operable, TAM heterogeneity is also the obstacle for TAM targeting drugs. Rather than bulk TAMs, targeting a key small portion of TAMs could be more effective with reduced side effects, which might be a future direction. Reprogramming macrophages towards antitumor phenotypes, rather than tumor suppressive ones, represents a promising direction, even though the potential for macrophage subset reprogramming has just been uncovered. Although TAM-targeting methods are still at the early stage, investigation into mechanisms of resistance to TAM-based immunotherapies is urgently needed as very limited data is available currently. The plasticity of macrophages allows them to switch phenotypes under different conditions, potentially contributing to drug resistance. Additionally, TAM-targeted cancer prevention and vaccine strategies should be considered, given the crucial roles of TAMs in cancer initiation, progression, and the formation of an immunosuppressive tumor microenvironment.

With the recent advancement of CAR-armed macrophage technology, its clinical potential still requires thorough evaluation through both preclinical and clinical trials. We would like to emphasize that the successful integration of CAR-macrophages with other therapies, such as CAR-T cells, in future clinical applications will depend on several key factors: (1) the ability of CAR-macrophages to sustain potent and long-lasting anti-tumor activity. As we know, one major issue with CAR-T cells in clinical applications is their tendency to become exhausted, leading to a loss of sustained functionality in some patients 10. Could CAR-M cells face similar challenges? (2) whether the toxicity and side effects associated with CAR-macrophages are manageable and potentially lower than those of CAR-T cells; and (3) the identification of additional tumor-specific surface antigens suitable for effective CAR-macrophage targeting.

The current TAM-targeting approaches face several limitations, and several challenges need to be addressed to better understand the roles of TAMs in cancer, including: (1) clarifying the tumor heterogeneity which may complicate the development of universal therapies targeting TAMs; (2) further understanding the complexity of TAM polarization, because TAMs can exist in a range of activation states (M1, M2, etc.), and this plasticity makes it challenging to target TAMs effectively without disrupting their beneficial roles in tissue homeostasis and immune regulation; (3) further understanding the molecular mechanisms that influence the function of TAMs, such as TAM-associated metabolites that promote tumor progression and TAM-specific transcriptional and epigenetic factors, as well as surface markers, to distinguish between pro- and anti-tumoral TAM subsets; (4) elucidating the detailed mechanisms underlying TAM-mediated immunosuppression in the tumor microenvironment, for example, how TAMs interact with other immune cells and tumor cells, and whether we use certain molecular signatures to predict the efficacy of therapies targeting TAMs?^242^ (5) developing novel delivery systems to enhance drug penetration for efficient targeting of TAMs; and (6) further understanding the resistance mechanisms of TAM-targeting therapies, for examples, the upregulation of alternative pathways or through the recruitment of other immune cells that compensate for TAM depletion or modulation.

## 6. Concluding remarks

In this review, we summarize the origins and polarization of tumor-associated macrophages (TAMs), discuss their role in regulating tumor development and immunity, and highlight the latest strategies in TAM-targeting cancer immunotherapy. The inherent heterogeneity of TAMs allows them to interact with various cells and participate in tumorigenesis and cancer immunity through diverse mechanisms, providing numerous opportunities for developing TAM-targeting therapies. However, for these strategies to be successfully translated into clinical practice, a more comprehensive and precise understanding of TAM heterogeneity and plasticity is essential. While several compounds, antibodies, and TAM engineering approaches have been developed, further supportive testing is needed to evaluate their clinical potential, both alone and in combination with other therapies, across different cancer contexts. Ongoing basic, translational, and clinical research will open new avenues for innovative therapeutic interventions, with promising outcomes expected in the future.

## Figures and Tables

**Table 1:** The different activators and biological functions of the M2 macrophage.

Subgroups	Upstream activators	Functions
M2a	IL-4, IL-13	Anti-inflammatory and tissue repair
M2b	IL-1β, TLR Ligands	Th2 activation and regulation of the immune response
M2c	IL-10, TGF-β, Glucocorticoids	Phagocytosis and immunosuppression
M2d	TLR Ligands, A2R agonists	Pro-tumor and angiogenesis

## Abbreviations

APOE: apolipoprotein E
bFGF: basic fibroblast growth factor
BM: bone marrow
CAR: chimeric antigen receptor
CCL: C-C motif chemokine ligand
ccRCC: clear cell renal cell carcinoma
CRC: colorectal cancer
CREM: CAMP responsive element modulator
CSF1R: colony stimulating factor 1 receptor
CTLA4: cytotoxic T-lymphocyte-associated protein 4
CTL: cytotoxic T lymphocyte
CXCL8: chemokine ligand 8
EGF: epithelial growth factor
EMT: epithelial-mesenchymal transformation
FOLR2^+^: folate receptor 2^+^
GPCR: G protein-coupled receptor
HBP: hexosamine biosynthetic pathway
HCC: hepatocellular carcinoma
HGF: hepatocyte growth factor
HIF-1α: hypoxia-inducible factor 1α
HLA-G: human leukocyte antigen G
HOXB13: homeobox B13
IFN: interferon
LAP: LC3-associated phagocytosis
LCN2: lipocalin-2
IL: interleukin
iNOS: inducible NO synthase
IRF4: interferon regulatory factor-4
LPMs: large peritoneal macrophages
LPS: lipopolysaccharide
MAO-A: monoamine oxidase A
MDSCs: myeloid-derived suppressor cells
MMPs: matrix metalloproteinases
MPLA: monophosphoryl lipid A
NFκB: nuclear transcription factor-κB
NK: nature killer
NOS: nitric oxide synthase
NRF1: Nuclear Respiratory Factor 1
NSCLC: non-small cell lung carcinoma
PDAC: pancreatic ductal adenocarcinoma
PDGF: platelet-derived growth factor
PD-L1: programmed cell death protein ligand 1
PD-1: programmed cell death protein 1
PITPNM3: phosphatidylinositol transfer protein 3
PS: phosphatidylserine
RCC: renal cell carcinoma
ROS: reactive oxygen species
STAT3: Signal transducer and activator of transcription 3
STING: stimulator of interferon response CGAMP Interactor 1
*SPP1*: secreted phosphoprotein 1
S1P: sphingosine-1-phosphate
TAA: tumor-associated antigens
TAMs: Tumor-associated macrophages
TIM4: T cell immunoglobulin and mucin domain-containing molecule-4
TLRs: toll-like receptors
TP: thymine phosphorylase
TGF-β: transforming growth factor β
TIMs: tumor-infiltrating monocytes
TME: tumor microenvironment
TNF-α: tumor necrosis factor-α
TREM2: triggering receptor expressed on myeloid cells 2
TRMs: tissue resident macrophages
VEGF: vascular endothelial growth factor
WGP: whole β-glucan particle
15-PGDH: 15-hydroxyprostaglandin dehydrogenase
